# The Effect of 3-Month Growth Hormone Administration and 12-Month Follow-Up Duration among Heart Failure Patients Four Weeks after Myocardial Infarction: A Randomized Double-Blinded Clinical Trial

**DOI:** 10.1155/2021/2680107

**Published:** 2021-01-23

**Authors:** Afshin Amirpour, Mehrbod Vakhshoori, Reihaneh Zavar, Hadi Zarei, Masoumeh Sadeghi, Behzad Yavari

**Affiliations:** ^1^Cardiac Rehabilitation Research Center, Cardiovascular Research Institute, Isfahan University of Medical Sciences, Isfahan, Iran; ^2^Heart Failure Research Center, Cardiovascular Research Institute, Isfahan University of Medical Sciences, Isfahan, Iran; ^3^Chamran Hospital, Department of Critical Care Nursing, School of Nursing and Midwifery, Isfahan University of Medical Sciences, Isfahan, Iran

## Abstract

**Background:**

The probable impact of growth hormone (GH) as a heart failure (HF) treatment strategy is still less investigated. Therefore, we aimed to evaluate the relation of 3-month GH prescription on left ventricular ejection fraction (LVEF), interventricular septum (IVS), posterior left ventricle (LV) thickness, end systolic and end diastolic diameters (ESD and EDD), and pulmonary arterial pressure (PAP) among Iranian individuals suffering from HF due to MI attack.

**Methods:**

A total of 16 clinically stable participants with HF diagnosis and LVEF < 40% were selected for enrollment in this pilot randomized double-blinded study. They were randomly assigned equally to groups received 5 IU subcutaneous GH or placebo. Injections were done every other day for a total of 3-month duration. After termination of intervention and nine months afterwards, cardiac outcomes were assessed.

**Results:**

Baseline and 12-month posttrial participants' characteristics were similar. LVEF was increased significantly by three months started from baseline in individuals receiving GH (32 ± 3.80% to 43.80 ± 4.60%, *P* = 0.002). During the next 9 months of follow-up concurrent with cessation of injections, LVEF was declined (43.80 ± 4.60% to 32.20 ± 6.97%, *P* = 0.008). LVEF and ESD were remarkably higher and lower in GH group compared with controls by the end date of injections (43.80 ± 4.60% vs. 33.14 ± 4.84%, *P* = 0.02 and 39.43 ± 3.45 mm vs. 33 ± 3.16 mm, *P* = 0.03, respectively). No other considerable association was found in terms of other predefined variables in neither GH nor placebo groups.

**Conclusions:**

GH administration in HF patients was associated with increased LVEF function. Several randomized clinical trials are necessary proving this relation. This trial is registered with IRCT201704083035N1.

## 1. Background

Heart failure (HF) is a disease resulting from any cardiac disorders leading to impaired ventricular filling or ejection fraction (EF) in a way that it poses one of the most common complications observed especially in patients in postischemic period [1]. Its prevalence has been reported to be ranged 1-12% and 0.5-6.7% in Western and Asian nations, respectively [2–4]. In USA, it was estimated that 400000 individuals would be diagnosed as new cases of congestive heart failure (CHF) annually, but annual hospital admission rates were approximately more than 700000 ones [5]. Imposing a great economic burden to governments for management of this chronic condition should be taken into account. It is reported that annual costs for caring patients suffering from CHF exceeds $10 billion [5]. Despite availability of angiotensin converting enzyme inhibitors (ACEIs) and *β*-blockers as cornerstones of CHF treatment, higher prevalence percentages mandate introduction of newer drugs [6]. One of the popular medications in this regard is growth hormone (GH). This endogenous hormone producing in pituitary gland has effects on most organs including heart via direct or indirect pathways with aid of insulin-like growth factor 1 (IGF-1) [7]. Several studies showed an affirmative effect on CHF both in humans and in animal experiments [8–11]. For instance, Fazio et al. reported that administration of GH to persons suffering from CHF significantly improved cardiopulmonary and exercise capacity [11]. Moreover, increased left ventricular (LV) mass and EF plus decreased in LV systolic volume was observed after receiving GH releasing peptide, named ghrelin [10]. Other distinct studies suggested the usefulness of GH administration on vascular functions and in decompensated situations [12, 13]. In spite of no definitive etiology, several possibilities have been suggested in order to explain these effects including cardiac myofilament sensitization to Ca^2+^, decreased rate of cardiac myocyte apoptosis, and peripheral vascular resistance. [7, 14] On the other hand, some researches failed to prove those favorable outcomes [6, 15]. Addition of GH to normal heart failure therapy did not show any significant effects on systolic and diastolic output or functional class [6]. Six months of GH treatment in 22 CHF individuals due to ischemia did not demonstrate any significant improvements in any LV functional components [15].

Due to these controversial results, the endpoints of this randomized clinical trial were defining left ventricular ejection fraction (LVEF), intraventricular septum (IVS), posterior LV wall thickness, and end systolic and end diastolic diameters (ESD and EDD) as well as pulmonary arterial pressure (PAP) after three months of GH administration in Iranian males suffering from CHF due to myocardial infarction (MI) in left anterior descending (LAD) coronary artery.

## 2. Materials and Methods

### 2.1. Study Design

This pilot randomized double-blinded controlled trial was performed from April 2016 till June 2018 in context of an ongoing project in one of the most popular governmental tertiary heart centers (Shahid Chamran hospital) located in Isfahan, Iran, in order to compare clinical outcomes of three-month GH administration to patients suffering from CHF. This study was approved by the ethical committee affiliated to Isfahan University of Medical Sciences (No. 395874) and was registered in Iranian Registry of Clinical Trials (IRCT201704083035N1).

### 2.2. Study Population

Any healthy male patients with no prior positive cardiac history aged as low as 40 to as high as 70 years with his first experience of MI due to single vessel LAD involvement with just one active plaque which had been fully revascularized afterwards leading to decreasing EF to less than 40% confirmed by two distinct echocardiography physicians at the time of discharge was eligible for being recruited in our study. We chose LAD due to higher prevalence of plaques in this artery in comparison to other major cardiac coronary arteries [16, 17]. Therefore, the likelihood of selecting eligible cases would be increased. Moreover, we just selected patients with one active plaque due to unawareness of effect of GH administration on other existed plaques. Even the presence of any of the following including insulin-dependent diabetes mellitus, respiratory diseases other than asthma, any chronic liver diseases, any renal problems leading to Cr ≥ 2.5 mg/dl or estimated glomerular filtration rate (eGFR) ≤ 60 ml/min/1.73m^2^, uncontrolled hypertension (HTN) within 4 weeks postdischarge, any systemic illnesses, infection with human immunodeficiency virus (HIV), any history of acute infections or antibiotic usages after discharge, corticosteroid or regular alcohol intake after hospital discharge, any thyroid or retinal disorders, presence of any positive findings in first degree or parents' history or physical examination indicative of malignancy or peripheral vascular disorders, any hematologic disorders even iron deficiency anemia, presence of musculoskeletal problems except osteoporosis and any cardiac disorders like severe valvular stenosis or regurgitation, moderate or severe ventricular hypertrophy, or any heart block excluded individuals from the study. Due to widespread predefined exclusion criteria and higher prevalence of cardiovascular events in men, we only recruited male participants to be able to find more study cases in this regard [18]. After selection of eligible participants by relevant questionnaire and laboratory investigations within 4 weeks after hospital discharge in addition to persistent lower LVEF of less than 40% diagnosed by echocardiography after one month post-MI attack, each individual was interviewed by the coordinator. Study process plus probable risks and benefits was explained, and the person had enough time making his decisions or asking any further questions. Finally, a written consent form was signed by each participant.

### 2.3. Study Intervention

The first participant was randomly assigned to either GH or control group by using computerized random numbers in a way that if the first number was odd, the subject would receive GH and placebo was given in terms of even number. Other assignments were done alternatively with 1 : 1 balanced randomization and form that time, and a three-month trial was initiated for that specified participant. Intervention group received 5 IU subcutaneous injection of GH (Omnitrope®, Austria) every other day for a total duration of 3 months. This dosage had been chosen based on national endocrine guidelines as well as previously published articles [19, 20]. Same volume of distilled water was filled in syringes and given to placebo groups with the exact duration and period interval as GH group. In order to minimize the likelihood of noncompliance, all injections were done by trained personnel. Every week, blood pressure (BP) and random blood sugar (BS) were assessed for each participant, and in terms of occurrence of any new coronary artery diseases or GH complications including BS ≥ 200 mg/dl, carpal tunnel syndrome, blurring vision, uncontrolled HTN defining as occurrence of HTN in individuals without prior history, or insuppressible BP in hypertensive participants previously controlled by appropriate anti-HTN agents, the subject was excluded from study and referred for treatment. The only group which was not blinded to this trial was nurses giving GH or placebo to participants.

### 2.4. Outcomes

The primary endpoint was assessing clinical outcomes resulting from GH administration on LVEF (%), IVS (mm), posterior LV wall thickness (mm), ESD (mm), EDD (mm), and PAP (mmHg). After completion of a three-month trial and the next nine months afterwards, 3-dimensional (3D) and 2-dimensional (2D) echocardiography (Phillips IE 33, Netherlands) was utilized measuring LVEF by biplane Simpson method and the other aforementioned variables, respectively, and the mean values were reported.

### 2.5. Sample Size

Considering 95% confidence interval (CI) and power of 80% plus possible proportion (P) of 0.5 and permissible error (d) of 25%, total required sample was estimated to be 32 which each group contained 16 individuals. During the project and due to lack of sample recruitment because of extensive exclusion criteria and financial issues as well as unawareness of probable GH interaction with inactive coronary artery plaques, the total number of required samples declined to 16 and equal subjects (*n* = 8) were allocated to GH and placebo group.

### 2.6. Statistical Analysis

Categorical and continuous variables were reported as frequency (percentage) and mean ± standard deviation (SD), respectively. Chi-square test was used in analyzing nominal variables. In order to compare numerical ones, Student's *t*-test was utilized. All analyses were done using Statistical Package for Social Sciences (SPSS Inc., version 20.0, Chicago, IL, USA), and *P* values less than 0.05 were considered statistically significant.

## 3. Results

**Figure 1:**
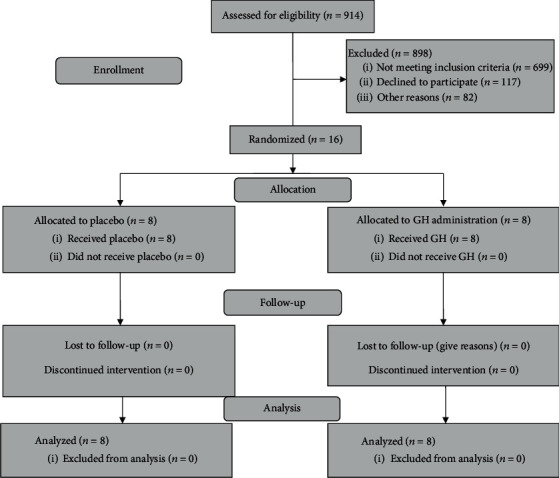
Flow diagram of trial profile.

After assessment for eligible individuals for enrollment in this study and discarding subjects due to different reasons including not meeting inclusion criteria, unwillingness for participation, or other personal reasons, 16 subjects were randomly assigned and allocated to distinct groups of GH (*n* = 8) or placebo (*n* = 8) (Figure 1). All participants completed the entire project period from April 2016 to the end of 12-month follow-up duration in June 2018. Data of all persons recruited in the study were available for intention-to-treat analysis. General characteristics of study population across different categories of intervention both at the baseline and at the end of follow-up duration have been shown in Table 1. Our findings showed that there was no significant relation in terms of any demographic properties, BP indices, or medication usages in neither two time frames.  Table 2 provides information on distribution of clinical cardiovascular outcomes at baseline, 3, and 12 months after study initiation according to GH or placebo treatment. Participants underwent GH treatment had significant increased LVEF within the first 3 months after start of intervention (32.3 ± 3.80 to 43.80 ± 4.60, *P* = 0.002). Nine months after termination of GH injection, considerable declined LVEF percentage had been observed (*P* = 0.008). Further analysis proved insignificant association from baseline to the end date of follow-up duration. There was no significant relation in terms of other clinical cardiovascular outcomes including IVS, posterior LV wall thickness, ESD, EDD, and PAP among GH group subjects. Although no considerable association had been found based on desired variables in placebo-group individuals, there was an exception showing significant decreased LVEF percentage between the time intervals from start to the end of follow-up (31.86 ± 4.18 to 24.33 ± 1.15, *P* = 0.02). Furthermore, our outcomes revealed that after 3 months concurrent with the date of injection ending, participants received GH had significantly higher percentages of LVEF compared to individuals taking placebo (43.80 ± 4.60 vs. 33.14 ± 4.84, *P* = 0.003). Participants received GH had significantly lower ESDs in comparison to controls after the injections were completed (39.43 ± 3.45 mm vs. 33 ± 3.16 mm, *P* = 0.03). None of our participants in neither GH group nor placebo group experienced any side effects during the implementation of this project.

## 4. Discussion

The current study is aimed at evaluating the relation between GH administration and clinical cardiovascular outcomes in Iranian HF patients due to LAD infarction with one active plaque. Our findings suggested that a 3-month injection of GH was associated with significant increased LVEF function and reduced ESD compared to placebo takers, but the raised LVEF was decreased after cessation of GH injections in the next 9 months. Since HF declines patients' quality of life and induces considerable economic problems, GH prescription might be categorized as a novel treatment strategy decreasing its prevalence and enhancing individuals' feeling of wellness.

### 4.1. Comparison with Previous Findings

Our findings were compatible with several studies [10, 13, 21, 22]. For instance, Nagaya and colleagues performed a study in order to evaluate the association of GH administration and cardiac outcomes. They recruited 10 clinically stable HF patients with LVEF < 35% and injected ghrelin (2 *μ*g/kg) twice daily by intravenous route for a total duration of 3 weeks. Furthermore, they enrolled 8 matched subjects nonrandomly as control group. Their final analysis revealed that participants receiving GH had significant elevated LVEF percentage (27 ± 2% to 31 ± 2%, *P* < 0.05) [10]. Also, a meta-analysis done by Le Corvoisier et al. suggested that GH therapy in chronic HF was associated with increased most of cardiac parameters including LVEF, IVS, and posterior LV wall thickness (+5.10 ± 1.74%, *P* < 0.05; +0.55 ± 0.43 mm, *P* < 0.001; and +1.01 ± 0.44 mm, *P* < 0.01, respectively) [21]. Even short-term treatment with GH showed improved LVEF outcomes. Twenty clinically stable HF patients because of coronary artery diseases were randomly assigned to low- and high-dose group. The former one received 5 *μ*g/kg of GH each day for the first four days and 10 *μ*g/kg for the next four days. The latter group received 10 *μ*g/kg and 20 *μ*g/kg with similar time frame. The results showed that active metabolite induced by daily 10 *μ*g/kg of GH administration was correlated positively with elevated LVEF (*r* = 0.59 and *P* = 0.006) [22]. Moreover, GH prescription in acute setting resulted in better outcomes. A total number of 6 unstable decongested chronic HF patients due to ischemic, idiopathic dilated, and peripartum cardiomyopathy consisting of four males and two females with median age of 51 years were enrolled in the study and received daily 8 IU of GH subcutaneously for median of 26 days. Their ultimate findings showed that LVEF percentage was significantly improved from median of 23 to 28% (*P* < 0.007) [13]. GH deficiency has also been suggested to negatively impact cardiac outcomes among individuals with HF. Arcopinto et al. implemented a cohort study in order to evaluate the prevalence of GH deficiency as well as probable cardiac findings in chronic HF patients. They recruited 130 patients and figured out that GH deficiency prevalence was 30%. They reported that patients with normal GH levels had significantly smaller EDV (-28%, *P* = 0.008) and ESV (-24%, *P* = 0.015) as well as reduced LV end systolic wall stress (-21%, *P* = 0.03). After median follow-up duration of 3.5 years, they found that all-cause mortality was more frequently observed among those patients with simultaneous chronic HF and GH deficiency [23]. Moreover, GH therapy on chronic HF patients with GH deficiency has been evaluated in several studies. Cittadini and colleagues selected 56 patients with concurrent HF and GH deficiency and randomly allocated them to intervention (*n* = 28) and control group (*n* = 28). The patients in the first group received 0.012 mg/kg GH every second day. Their final findings suggested that LVEF was significantly increased from 34 ± 2% to 36 ± 2% (*P* < 0.01) [24]. An extension of the aforementioned randomized controlled single-blinded trial showed that among 17 patients related to GH group and 14 patients in control group who completed the follow-up duration of 4 years, LVEF was remarkably elevated by 10 ± 3% in those received GH. On the other hand, controls had reduced LVEF by 2 ± 5% [25]. Also, findings of a review article indicate that GH prescription effectively increases exercise tolerance and reverse LV remodeling [26]. On the other hand, other studies' results were in disagreement with ours [6, 15, 27, 28]. Isgaard and colleagues recruited 22 individuals suffering from congestive HF due to idiopathic dilated cardiomyopathy, ischemic heart disease, or valvular surgery and allocated them to either placebo (*n* = 11) or GH (*n* = 11) group. All participants in the intervention group received GH with a total weekly initial dosage of 0.1 IU/kg for the first week and 0.25 IU/kg for the next weeks which had been injected each day. After 3 months, their outcomes showed that there was not any significant improvement in cardiac functions. Their outcomes should be cautiously interpreted due to their sample size and daily administration of GH [6]. The results of a double-blinded randomized clinical trial on 19 congestive HF subjects with LVEF of less than 30% declared that 8-week treatment with 0.03 U/kg of GH injected daily insignificantly improved LVEF compared to individuals receiving placebo. Their outcomes might be affected by short therapy duration and probable low-dose agent administration [27]. Moreover, 20 persons suffering from postischemic chronic HF (LVEF < 40%) were divided equally to groups getting GH or placebo for a period of 6 months. They concluded that although participants felt subjective sense of well-being more frequently, adding GH to conventional HF treatment did not improve cardiac functions. Self-administered injection by participants themselves might affect their findings especially due to their long duration of intervention [28]. Smith and colleagues recruited 22 patients with mean age of 64 years and diagnosis of ischemic HF having LVEF < 40%. Participants allocated to GH group nonblindly receiving self-administered subcutaneous GH each day with titration up to final dosage of 2 IU for six months. They found that LVEF did not improve significantly with aid of GH prescription. Chronicity of HF and nonblinded design of intervention could be potential explanations for their results [15].

### 4.2. Pathophysiological Hypothesis

In spite of unknown exact etiology in terms of GH administration and cardiovascular outcomes, several theories have been postulated. One of the possibilities could be due to direct effect of GH on intracellular Ca^2+^ located in cardiac muscle cells leading to enhancement in heart contractility capability. Furthermore, this anabolic hormone has been suggested to decrease peripheral vascular resistance resulting in improvement in hemodynamic status [7, 29, 30]. Increased cardiac myofibril sensitivity to Ca^2+^ or myosin isoform change has been suggested as other probable mechanisms [14, 31–33].

One of the most remarkable strength of this current article was a long period of follow-up without any sample missing or drug side effects. We tried to define the exclusion criteria with multiple components in order to select subjects with almost exact similar characteristics in both intervention and control groups. According to best knowledge, this study was the first in literature administrating GH to clinically stable HF patients after a quite short period of MI with predefined follow-up duration of 12 months. In addition to GH injections done by trained personnel in order to decrease probable self-administered bias, all administrations were performed every other day. This method has been suggested to be superior over daily injections, because pulsatile GH plasma concentration induces more IGF-1 production in cardiac cells [34].

### 4.3. Study Limitations

This study was not free from limitations. We did not assess GH status of study population to find the GH deficiency prevalence. IGF-1 levels were not measured to observe any improvement in GH action in peripheral effectors or confirm GH injections, but the method in which trained health care professionals had been selected for drug administration could be probably compensate the latter limit. Cardiovascular performance of patients was not assessed using different available tests including cardiopulmonary exercise testing (CPET) or 6-minute walk test (6MWT) due to unknown mechanism of GH effect on other coronary plaques. Furthermore, we just enrolled only male individuals suffering from HF due to just ischemic attacks; therefore, our findings should be generalized with cautious to the opposite gender and HF patients with other prior etiologies. Restriction of financial aids leading to quite small sample size could be considered as another disadvantage.

In conclusion, we found that a 3-month GH administration in HF patients after MI attack has been associated with increased percentage of LVEF. Consequently, this treatment fashion could be prescribed as a safe modality in individuals experiencing HF shortly after ischemic attack. Several randomized double-blinded clinical trials are required confirming these associations.

## Figures and Tables

**Table 1:** Characteristics of study participants across different categories of intervention or control group at baseline and 12 months after study initiation.

			GH group (*n* = 8)	Placebo group (*n* = 8)	*P* value
Baseline	Age (years)		56.2 ± 2.1	51.7 ± 3.2	0.21
	BMI (kg/m^2^)		25.2 ± 4.6	24.9 ± 4.8	0.64
	Education level (≥diploma)		87.5	100	0.93
	Occupation status	Employee	62.5	50	0.48
		Retired	37.5	50	
	Blood pressure (mmHg)	Systolic	128.4 ± 10.2	126.7 ± 9.9	0.09
		Diastolic	78.2 ± 5.4	80.3 ± 6.1	
	Hemoglobin (g/dl)		12.8 ± 1.4	13.1 ± 1.2	0.51
	Cholesterol (mg/dl)		167.7 ± 32.5	165.2 ± 28.4	0.32
	Triglyceride (mg/dl)		144.3 ± 47.3	143.6 ± 45.6	0.59
	LDL-C (mg/dl)		95.3 ± 17.9	94.8 ± 16.8	0.66
	HDL-C (mg/dl)		45.2 ± 8.8	43.4 ± 7.9	0.18
	eGFR (ml/min/1.73m^2^)		0.86 ± 0.13	0.86 ± 0.12	0.88
	Medication usage (%)	ACEI/ARB	100	100	0.18
		*β*-Blocker	100	100	
		COX-inhibitors	100	100	
		HMG COA reductase inhibitor	100	100	
		Thienopyridines	100	100	

After 12 months	Age (years)		58.3 ± 2.3	52.8 ± 5.1	0.13
	Weight (kg)		80.0 ± 5.0	83.8 ± 5.8	0.38
	BMI (kg/m^2^)		25.8 ± 1.9	27.4 ± 1.7	0.28
	Blood pressure (mmHg)	Systolic	120.6 ± 29.1	115.8 ± 14.2	0.75
		Diastolic	83.0 ± 15.1	78.4 ± 12.7	0.65
	Hemoglobin (g/dl)		13.5 ± 1.4	13.3 ± 1.8	0.85
	Cholesterol (mg/dl)		149.3 ± 33.5	148.2 ± 28.2	0.66
	Triglyceride (mg/dl)		143.4 ± 32.8	145.3 ± 38.7	0.75
	LDL-C (mg/dl)		91.3 ± 20.4	88.3 ± 21.3	0.38
	HDL-C (mg/dl)		44.2 ± 8.8	45.1 ± 7.2	0.59
	eGFR (ml/min/1.73m^2^)		0.84 ± 0.13	0.85 ± 0.14	0.56
	Medication usage (%)	ACEI/ARB	100	100	1.00
		*β*-Blocker	100	100	
		COX-inhibitors	100	100	
		HMG COA reductase inhibitor	100	100	
		Thienopyridines	100	100	

GH: growth hormone; BMI: body mass index; LDL-C: low-density lipoprotein cholesterol; HDL-C: high-density lipoprotein cholesterol; eGFR: estimated glomerular filtration rate; ACEI: angiotensin converting enzyme inhibitor; ARB: angiotensin receptor blocker; COX-inhibitor: cyclooxygenase inhibitor; HMG-CoA: 3-hydroxy-3-methyl-glutaryl-CoA.

**Table 2:** Distribution of clinical cardiovascular outcomes at baseline, 3, and 12 months after study initiation across different categories of intervention or control group.

Endpoint	GH group (*n* = 8)			*P* ^∗^	*P* ^†^	*P* ^‡^	Placebo group (*n* = 8)			*P* ^∗^	*P* ^†^	*P* ^‡^	*P* ^¶^	*P* ^ƪ^	*P* ^ƛ^
	Baseline	After 3 months	After 12 months				Baseline	After 3 months	After 12 months						
LVEF (%)	32 ± 3.80	43.80 ± 4.60	32.20 ± 6.97	0.002	0.008	0.95	31.86 ± 4.18	33.14 ± 4.84	24.33 ± 1.15	0.25	0.05	0.02	0.95	0.003	0.06
IVS thickness (mm)	9.40 ± 1.14	9.20 ± 1.09	7.40 ± 2.07	0.70	0.10	0.06	7.57 ± 1.81	8.14 ± 1.86	8 ± 0	0.10	0.05	0.42	0.07	0.28	0.55
Posterior LV wall thickness (mm)	9.20 ± 1.09	9 ± 1.22	8 ± 1.73	0.37	0.47	0.38	8.86 ± 1.21	8.86 ± 0.90	8.33 ± 1.15	0.99	0.18	0.18	0.62	0.82	0.78
End diastolic diameter (mm)	54.14 ± 4.67	53.86 ± 3.02	63 ± 7.81	0.44	0.21	0.76	53.60 ± 4.39	50.80 ± 3.70	58 ± 6.12	0.77	0.55	0.34	0.53	0.43	0.76
End systolic diameter (mm)	39.57 ± 3.10	39.43 ± 3.45	51 ± 6.08	0.78	0.56	0.44	35.80 ± 6.01	33 ± 3.16	42.80 ± 10.91	0.23	0.39	0.60	0.76	0.03	0.32
Pulmonary arterial pressure (mmHg)	24.3 ± 2.1	23.4 ± 1.9	23.7 ± 2.2	0.32	0.85	0.22	25.4 ± 2.2	25.1 ± 1.6	24.5 ± 2	0.46	0.77	0.55	0.50	0.81	0.51

GH: growth hormone; LVEF: left ventricular ejection fraction; IVS: interventricular septum; LV: left ventricle. ^∗^*P* value between baseline and after 3 months. ^†^*P* value between after 3 months and after 12 months. ^‡^*P* value between baseline and after 12 months. ^¶^*P* value between growth hormone and placebo groups at baseline. ^ƪ^*P* value between growth hormone and placebo groups after 3 months. ^ƛ^*P* value between growth hormone and placebo groups after 12 months.

## Data Availability

The datasets generated during and/or analyzed during the current study are not publicly available due to confidential issues but are available from the corresponding author on reasonable request.

## Abbreviations

MI: Myocardial infarction
GH: Growth hormone
HF: Heart failure
LVEF: Left ventricular ejection fraction
IVS: Interventricular septum
LV: Left ventricle
EF: Ejection fraction
CHF: Congestive heart failure
ACEIs: Angiotensin converting enzyme inhibitors
IGF-1: Insulin-like growth factor 1
LAD: Left anterior descending
GFR: Glomerular filtration rate
HTN: Hypertension
HIV: Human immunodeficiency virus
BP: Blood pressure
BS: Blood sugar
3D: 3-dimensional
2D: 2-dimensional
CI: Confidence interval
SD: Standard deviation
SPSS: Statistical Package for Social Sciences
CPET: Cardiopulmonary exercise testing
6MWT: 6-minute walk test.
